# Ultrasound detects synovitis in replaced and other surgically operated joints in rheumatoid arthritis patients

**DOI:** 10.1186/s41927-019-0107-2

**Published:** 2020-02-03

**Authors:** Evan A. Choate, Gurjit S. Kaeley, Jenny Brook, Roy D. Altman, John D. FitzGerald, Astrid R. Floegel-Shetty, David A. Elashoff, Veena K. Ranganath

**Affiliations:** 1 Department of Medicine, Division of Rheumatology, University of California, Los Angeles, CA USA; 2 College of Medicine, University of Florida, Jacksonville, Florida USA; 3 Department of Medicine/Biostatistics, Division of General Internal Medicine and Health Services Research, University of California, Los Angeles, CA USA

**Keywords:** Arthritis, rheumatoid, Synovitis, Ultrasonography, Surgical procedures operative, Joints, Response

## Abstract

**Background:**

Joint replacements continue to occur during a rheumatoid arthritis (RA) patient’s lifetime despite significant advances in available treatment options. The purpose of this study was to examine and quantify synovitis in surgically operated joints by ultrasound (US) in RA patients starting a new therapeutic agent.

**Methods:**

RA subjects were enrolled in either tocilizumab or tofacitinib open-label, investigator-initiated trials and were assessed by ultrasound. In a subset of RA patients with joint replacements and/or operations of joint areas (OJA; e.g. joint arthroscopies, fusions, and synovectomies), joint-level scores of synovitis were compared between replaced joints, OJAs, and native joints. Joint-level synovitis was measured by grayscale (GSUS (0–3)) and power Doppler (PDUS (0–3)) at baseline and follow-up (3–6 months). McNemar’s test or Wilcoxon signed rank test utilized the mixed effects ordinal logistic regression models.

**Results:**

Twenty RA patients had a total of 25 replaced joints and 24 OJA. All replaced joints had GSUS> 1 and 92% had PDUS> 1 at baseline, while OJA and native joints had lower evidence of GSUS> 1 (37.5, 38% respectively) and PDUS> 1 (45.8, 62% respectively). GSUS and PDUS semiquantitative scores improved significantly with treatment in replaced joints (*p* = 0.01, *p* = 0.007), and native joints (*p* < 0.001 both), but not OJA.

**Conclusions:**

In RA, joint replacement does not eliminate or prevent ultrasound measured synovitis, where all replaced joints have some evidence of US synovitis. US can also act as a potential marker of response to therapy in replaced joints. Scoring US synovitis in replaced joints should be considered in ultrasound RA clinical trials.

**Trial registration:**

ClinicalTrials.gov NCT01717859 (registered 10/31/2012); ClinicalTrials.gov NCT02321930 (registered 12/22/2014).

## Background

Joint replacements in patients with rheumatoid arthritis (RA) occurs due to worsening patient-reported pain and function, progression of radiographic joint damage, high disease activity, and elevated acute phase reactants [1]. The increased early use of potent disease-modifying anti-rheumatic drugs (DMARDs) is thought to delay or even prevent joint replacement [2]. While this approach has significantly reduced the number of replaced joints in RA patients over the last several decades, joint replacement surgeries occur in up to 34% of RA patients with 30 years of disease duration, most commonly involving hip and knee [3].

Musculoskeletal ultrasound (MSUS) is an American College of Rheumatology (ACR) and European League Against Rheumatism endorsed imaging modality for evaluating synovitis and synovial hypertrophy in RA [4, 5]. There is known difficulty in accurately assessing inflammation of replaced joints by magnetic resonance imaging or other imaging modalities due to distortions and artifacts, thus MSUS may fulfill this unmet need. Ultrasound grayscale (GSUS) and Power-Doppler (PDUS) modes are sensitive methods for detecting and measuring synovitis [6]. Both help track and predict the progression of joint destruction and response to RA treatment, with recent work suggesting that increased baseline PDUS may identify modifiable disease activity [5, 7]. Elevated baseline PDUS may also predict RA patients who will respond to therapy [8]. MSUS activity has not yet been systematically evaluated in RA patients with joint surgeries.

We performed a post-hoc analysis of a subset of RA patients with prior joint surgeries enrolled in one of two open-label therapeutic trials, utilizing MSUS to measure synovitis. The purpose of this study was to quantify ultrasound synovitis of surgically operated joints in RA patients starting a new therapeutic agent.

## Methods

### Patients and study design

Two open-label investigator-initiated clinical trials (NCT01717859, NCT02321930) recruited RA patients from two university-based rheumatology clinic sites following institutional review board approval (IRB#12–001547, IRB#14–001148) and appropriate patient consent obtained. While the primary endpoint of these two studies aimed to examine early changes in PDUS, this post hoc study aimed to evaluate a subset of RA subjects who underwent prior joint surgery and quantify ultrasound synovitis of these operated joints. Participants’ joints were pooled into a single cohort and characterized as replaced joints, operated joint areas (OJA), or native joints (no prior surgery). OJA included joint arthroscopies, joint fusion, synovectomies, and tendon surgeries; these interventions were grouped together given their individual small sample sizes. Carpal tunnel surgeries and nerve transpositions were excluded. Patients at baseline were at least 18 years of age, met 1987 ACR criteria for RA, demonstrated disease activity score/erythrocyte sedimentation rate (DAS28/ESR-4 item) ≥ 3.2, and had cumulative Power Doppler score > 10 over 32 joints (see below for scoring). Patients also had received ≤10 mg prednisone and maintained stable concomitant DMARDs for at least 1 month. The 6-month tocilizumab study began with an infusion of 4 mg/kg of drug every 4 weeks and escalated to 8 mg/kg if DAS28/ESR-4 item was > 3.2 at 12 weeks, with patient and sonographer blinded to dosage step-up. The 3-month tofacitinib trial administered 5 mg of drug twice-daily by mouth. Only patients with replaced joints and/or OJA that completed the trial were included for analysis.

### Ultrasound assessments

MSUS scanning was performed for tocilizumab patients (baseline and months 1, 3, 4, 6) and tofacitinib (baseline, 2 weeks, and 3 months) to assess disease activity and synovitis at pre-specified joints. MyLab70C US machine (Biosound Esaote, Fishers, IN) was used for image acquisition in the tocilizumab trial (12–18 MHz linear probe), whereas tofacitinib images were obtained using GE LogicE9 US machine (GE Healthcare, Chicago, IL) (6–15 MHz linear probe), as mandated in the respective parent clinical trials. MSUS assessments were conducted by one of two independent, experienced sonographer-rheumatologists by enrollment site (GK, VKR).

Sonography of some joints can be challenging due to lower incidence of involvement, lack of standardization of the optimal views in RA, and increases in depth that decrease sensitivity of PDUS detection. As such, there is no consensus on the number of joints to scan in RA [9]. Our ultrasound protocol included bilateral GSUS and PDUS images of 16 joints commonly assessed in other RA MSUS studies: dorsal long, dorsal short, and volar long views of metacarpophalangeal (MCP) 1–5, proximal interphalangeal (PIP) 2–5, and interphalangeal (IP); dorsal long midline views of radiocarpal-intercarpal wrist joints and dorsal long and short views of radioulnar wrist joints (wrist); dorsal long views metatarsophalangeal (MTP) 2–5; and medial/lateral parapatellar axial oblique views of the knees. B-mode scanning of replaced joints and OJA, including joint position and depth, were similar to that performed on native joints. In subjects with joint replacement, hardware artifacts could be recognized as well as material in the pseudocapsule. In most circumstances, Doppler signal was present within the visualized intracapsular material. Joints that could not be assessed by ultrasound (e.g. severe anatomical deformation) were excluded from the MSUS joint-level analyses.

Each joint view was scored on a previously standardized semiquantitative scale ranging from 0 to 3 [10, 11]. The maximum score of all views was selected for each joint. Images were de-identified to patient and date and scored independently by two experienced sonographer rheumatologists by enrollment site (GK, VKR) who were blinded to the sequence of visits, the patient, and the clinical assessment. PDUS inter-rater reliability was 0.77, and intra-rater reliability ranged from 0.82–0.89 (weighted Kappa). GSUS inter-rater reliability was 0.57, and intra-rater reliability ranged from 0.65–0.76 (weighted Kappa).

### Clinical examinations

To capture clinical correlates for joints within and outside of the ultrasound protocol, a comprehensive, 68-joint count for tenderness and 66-joint count for swelling was collected by established convention at each visit and scored as absent (0) or present [1] for each joint. The clinical assessors were blinded to US data. A subset of 28 joints from these assessments also facilitated calculation of patient-level disease activity measures (DAS28 and Clinical Disease Activity Index [CDAI]). Replaced joints were excluded when calculating the DAS28/ESR-4 item and the CDAI. Joints not assessed for tenderness or swelling were excluded from joint-level analyses but were input as a score of zero for patient-level joint counts.

### Statistical analysis

Measures of disease activity were computed at the patient- and joint-level. Joint-level GSUS and PDUS measures were compared between baseline and follow-up time points using Wilcoxon signed rank tests. McNemar’s test was used to compare joint-level dichotomous measures of PDUS≥1, GSUS≥1, tenderness, and swelling. Logistic regressions for PDUS≥1, GSUS≥1, tenderness, and swelling, adjusted for anatomical joint site, were used to verify that the models above were not affected by different distributions of anatomical joint sites across the three joint types. Mixed effects ordinal logistic regression models were used to compare joint-level GSUS and PDUS scores over time between joint types. These models included terms for joint type, time, and the time by joint type interaction. A random effect term was also included to account for clustering of joints within subjects. Spearman correlation was used to test for association between the time since surgical procedure and both the baseline and end-point PDUS and GSUS scores. In addition, subgroup sensitivity analysis was performed for each of the clinical trials (tocilizumab vs tofacitinib), as well as for small vs large/medium (wrists, knees [ultrasound was not performed on shoulders, hips, elbows and shoulders]) joints to examine differential effects.

## Results

### Demographic and baseline characteristics of the patient cohort

Twenty RA patients with baseline and final visit data for analysis had prior joint surgeries. However, 16/20 patients had both baseline and final visit ultrasound scores and 18/20 patients had both for clinical joint count assessments. At baseline the cohort on average was 60.5 years of age (SD = 11.7) with an average disease duration of 15.2 years (SD = 11.0). The mean time elapsed since surgical manipulation was 13.1 years (SD = 11.3). Patients were 85% female, 55% Caucasian, and 30% African-American. The cohort included 95.3% seropositive patients, where 70% of patients were rheumatoid factor (RF) positive and 90% anti-cyclic citrullinated peptide (anti-CCP) positive. Mean baseline DAS28/ESR-4 item was 6.20 (SD = 0.88) and CDAI was 36.8 (SD = 10.3), indicating that the average patient experienced severe disease activity.

### Ultrasound assessment of replaced joints, OJA, and native joints

**Fig. 1:**
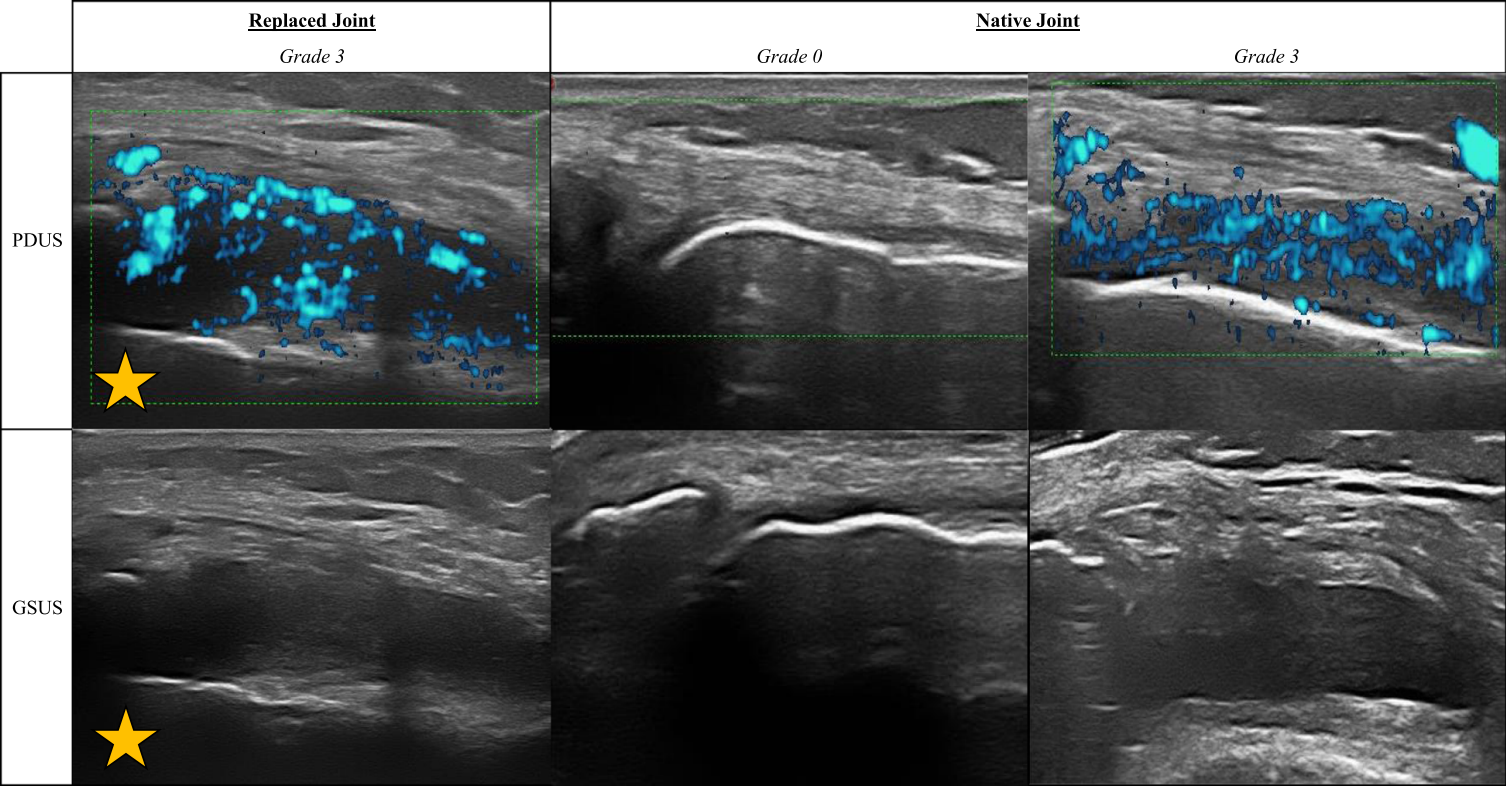
Synovitis detectable by ultrasound of the lateral knee. Left column, Power Doppler ultrasound (PDUS) and grayscale ultrasound (GSUS) of replaced joint, Grade 3 (severe synovitis). Middle column, PDUS and GSUS of native joint, Grade 0 (no synovitis). Right column, PDUS and GSUS of native joint, Grade 3 (severe synovitis). Yellow star indicates the location of the prosthesis

As stated above, sixteen RA patients each had 32 joints assessed by ultrasound: 25 replaced joints (14 MCP, 1 PIP, and 10 knee), 24 OJA (5 MCP, 6 wrist, 11 MTP, and 2 knee), and 463 native joints (141 MCP, 127 PIP, 32 IP, 26 wrist, 117 MTP, and 20 knee). Figure 1 shows images of grade 3 synovitis by PDUS and GSUS for lateral knee views in replaced joints and native joints.

At baseline, 92% of replaced joints, 37.5% of OJA, and 38% of native joints had PDUS > 1 (Table 1). Similar values, though numerically higher, were seen for joints with GSUS> 1 (100% replaced joints, 45.8% OJA, 62% native joints). Native joints exhibited a significant mean reduction in PDUS synovitis scores between baseline and final visit from 0.77 (SD = 1.11) to 0.54 (SD = 0.94) (*p* < 0.0001) and a decline in percent of native joints identified as PDUS≥1 from 38.0 to 29.2% (*p* = 0.0002) was observed. In the same interval, replaced joints demonstrated a reduction in mean PDUS scores from 2.28 (SD = 0.84) to 1.56 (SD = 1.16) (*p* = 0.007) and mean GSUS scores from 2.28 (SD = 0.54) to 1.72 (SD = 1.06) (*p* = 0.01); similarly, the percentage of replaced joints with PDUS> 1 and GSUS> 1 reduced significantly, from 92 to 72% and 100 to 80%, respectively (both *p* < 0.03). OJA showed a non-significant reduction in PDUS scores between baseline and final, from 0.92 (SD = 1.28) to 0.75 (SD = 1.11). Models adjusted for the anatomical joint site did not show differences in the results presented above (results not shown).

**Table 1:** Therapeutic response to bDMARDs or small molecule therapy in individual joints by surgery type. Joints were subdivided as native joints, surgically replaced, or operated joint areas. Disease activity at baseline and at study termination (3–6 months) were tracked clinically (18 patients) and by ultrasound measures (16 patients). Power Doppler ultrasound (PDUS); grayscale ultrasound (GSUS)

**Native Joints**						
		Baseline		End of Study		*p*-value
	*N joints*	*% Affected*		*% Affected*		
Tender (0–1)	1156	35.7		26.0		< 0.0001*
Swollen (0–1)	1127	24.8		13.0		< 0.0001*
% PDUS ≥1	463	38.0		29.2		0.0002*
% GSUS ≥1	463	62.0		57.0		0.045*
	*N joints*	*Mean (SD)*	*Median (IQR)*	*Mean (SD)*	*Median (IQR)*	
PDUS (0–3)	463	0.77 (1.11)	0 (0–2)	0.54 (0.94)	0 (0–2)	< 0.0001*
GSUS (0–3)	463	1.18 (1.10)	1 (0–2)	0.99 (1.01)	1 (0–1)	< 0.0001*
**Replaced Joints**						
	*N joints*	*% Affected*		*% Affected*		
Tender (0–1)	25	28.0		32.0		0.56
Swollen (0–1)	22	54.6		45.5		0.41
% PDUS ≥1	25	92.0		72.0		0.03*
% GSUS ≥1	25	100		80.0		0.03*
	*N joints*	*Mean (SD)*	*Median (IQR)*	*Mean (SD)*	*Median (IQR)*	
PDUS (0–3)	25	2.28 (0.84)	2 (2–3)	1.56 (1.16)	2 (0–2)	0.007*
GSUS (0–3)	25	2.28 (0.54)	2 (2–3)	1.72 (1.06)	2 (1–2)	0.01*
**Operated Joint Areas**						
	*N joints*	*% Affected*		*% Affected*		
Tender (0–1)	36	41.7		44.4		0.74
Swollen (0–1)	36	36.1		38.9		0.80
% PDUS ≥1	24	37.5		37.5		0.99
% GSUS ≥1	24	45.8		37.5		0.32
	*N joints*	*Mean (SD)*	*Median (IQR)*	*Mean (SD)*	*Median (IQR)*	
PDUS (0–3)	24	0.92 (1.28)	0 (0–2)	0.75 (1.11)	0 (0–1)	0.28
GSUS (0–3)	24	1.04 (1.23)	0 (0–2)	0.71 (0.95)	0 (0–2)	0.12

*McNemar’s test used for tender, swollen, PDUS≥1, and GSUS≥1 frequencies, Wilcoxon signed rank test used for PDUS (0–3) and GSUS (0–3)

**Fig. 2:**
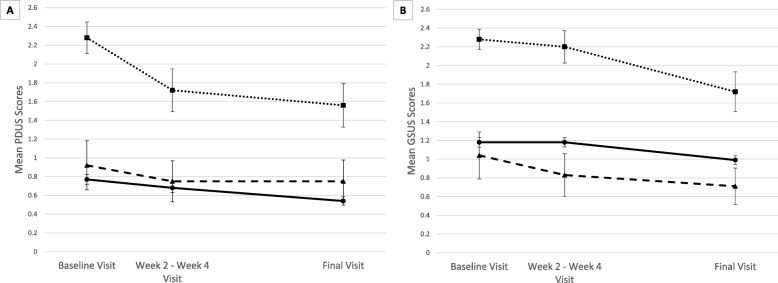
Mean synovitis scores for replaced joints, operated joint areas (OJA), and native joints. From baseline to final visit (*N* = 16 patients). Panel (**a)** measures the change in mean Power Doppler ultrasound (PDUS) scores over time. Panel (**b**) depicts the change in mean grayscale ultrasound (GSUS) scores over time. Legend: replaced joints (••■••), OJA (**—**▲**—**), and native joints (**—**●**—**)

The joint-level GSUS and PDUS scores evaluated over time differed between joint type groups (replaced joint, OJA, and native joint) in a mixed effects model (*p* < 0.001) (Fig. 2). The interaction between joint type and time was not significant in this model. There was no significant correlation between time since surgery and baseline PDUS score (r = 0.05) or GSUS score (r = 0.17); neither was there a significant correlation between time since surgery and response at the end of study (PDUS r = 0.16, GSUS r = 0.04).

Lastly, the subgroup sensitivity analyses performed were largely consistent between the two drugs and between joint types. PDUS and GSUS significantly improved between baseline and final assessment in naïve joints in both studies (*p* < 0.01 for all comparisons). The magnitude of improvement was also similar (ex. mean delta PDUS values of 0.23 and 0.3). In addition, we found similar findings across study drugs for replaced joints (PDUS improvement of 0.65, *p* = 0.05; and 0.8, *p* = 0.06). Similar findings were seen in drug subsets of OJA to the overall OJA results. Our comparisons between joint types found small and large joints experienced similar and significant decreases in PDUS for both in naïve (*P* < 0.001 for both small and large) and small replaced joints (*p* = 0.003) but not significantly for large replaced joints (*p* = 0.5) or small or large OJAs (*p* = 0.13 and 0.99 respectively).

### Clinical assessment of replaced joints or operated joint areas

A total of 18 RA patients each had 68 joints clinically assessed for tenderness: 25 replaced joints, 36 OJA, and 1156 native joints. The 18 RA patients were also were also clinically assessed for swelling at 66 joints: 22 replaced joints, 36 OJA, and 1127 native joints. There were 7 joints not examined for tenderness and 3 joints not examined for swelling, all of which were reported as missing data. Replaced joints and OJA did not demonstrate a significant tender or swollen joint response. However, for the native joints, the proportion that were tender improved significantly (35.7 to 26.0%, *p* < 0.0001), as did the proportion that were swollen (24.8 to 13%, *p* < 0.0001) (Table 1)**.**

## Discussion

We demonstrated that in patients with RA, measurable synovitis by MSUS exists in surgically operated joints, particularly in the area around replaced joints. Power doppler signal was seen in 92% of replaced joints, and grayscale-detected synovitis was seen in all replaced joints. In addition, we have shown that synovitis in replaced joints can be measurably responsive to therapy.

Despite reduced rates of arthroplasties due to early intervention with DMARDs, about a third of RA patients still require joint surgeries within 30 years of diagnosis, most commonly in the large joints of the hip and knee [2, 3]. To our knowledge, no studies have comprehensively characterized features of synovitis of RA replaced joints by sensitive and objective imaging technologies like MSUS, let alone by clinical exam. The extent of potentially clinically apparent chronic residual inflammation in this subset of joints has been left unrecognized and poorly understood. At present, clinical trials and other standard longitudinal assessments exclude replaced joints from the tender joint and swollen joint counts for the calculation of RA disease activity scores. However, operated joint areas are not excluded from joint counts. Our data suggest that replaced joints ought to be considered as part of the RA ultrasound joint examination, considering their elevated disease activity and responsiveness to treatment.

At the trial onset, no replaced joint was devoid of baseline synovitis, unlike native joints which presented with synovitis less commonly and with lesser severity. This trend extended throughout the 3–6 month study window. Replaced joints may show chronic subclinical levels of inflammation against the exogenous prosthetic that stimulate local osteolysis, [12] contain hyperactive synovium incompletely removed during arthroplasty, or experience recovery of the synovium through a separate unknown mechanism. Patients with replaced joints have been known to exhibit spikes in C-reactive protein and ESR levels postoperatively in healthy and RA cohorts before returning to preoperative baseline [13, 14]. Other work with FDG-PET technology demonstrates persistent disease activity in a 12-week postoperative period [15]. Our imaging findings support the notion that joint inflammation consistent with RA extends after surgical interventions that is readily quantifiable by ultrasound.

In addition, replaced joints exhibit a reduction in synovitis by study conclusion, as evidenced by significant improvements in GSUS and PDUS scores which mirrored that of native joints. These previously unrecognized phenomena further suggest that this abundance of pro-inflammatory mediators seen after replacement is amenable to treatment like in native joints, helping defy the common-held assumption that RA joints after surgery are unreliable and unresponsive targets for therapy. One case study using MSUS found that 3-month treatment with certolizumab pegol plus methotrexate reduced MSUS measures of inflammation in a knee status-post total arthroplasty, an outcome that further supports our study’s conclusions [16]. However, a difference in inflammation between surgery types was still seen by the end of study – with replaced joints demonstrating higher synovitis scores (PDUS: 1.56, and GSUS: 1.72) than native joints’ baseline (PDUS: 0.77, and GSUS: 1.18). The clinical implications of MSUS synovitis is unclear for the replaced joint, since only 28% were tender and 55% were swollen at baseline. Perhaps at the joint level, synovitis seen on ultrasound may not provide clinical value in the asymptomatic patient with joint replacement. On the other hand, it is known that *total* PDUS and GSUS scores improve with RA treatment, and these total MSUS synovitis scores with the addition of replaced joint MSUS synovitis scores may improve the detection of response to therapy. Future studies are still needed to quantify the added value to clinical trials of replaced joints in global RA outcome metrics and treatment considerations.

No significant change was seen in joint tenderness or swelling in either surgical joint cohort. A lack of reduced tenderness response may reflect local hyperalgesia driven by pro-inflammatory cytokines, central sensitization, or co-morbidities like fibromyalgia with RA or surgical intervention [17]. Similarly, while baseline GSUS and PDUS values as well as their downward trend over time in OJA resembled those of native joints, no statistical significance was seen. OJAs represent a heterogenous population most directly due to the specific surgical operation performed, which this current study not powered to stratify. However, it is possible that the less invasive procedure inflicted upon these joints compared to replaced joints may reflect less severe prior disease activity. Interestingly these are joints examined for tenderness/swelling to include in RA joint count disease activity assessments; should OJA in fact be unresponsive to therapy as our small sample posits, it is worth exploring whether these joints add value to such assessments of drug efficacy and response in clinical trials.

This study was not without limitations. Due to a limited sample size, clinical joint swelling and tenderness improvement may not have been seen in replaced joints and OJA. Perhaps with a larger cohort of OJA, we may have also seen significance in response to therapy by MSUS. The MSUS protocol followed only assessed 32 joints and did not require the scanning of other more commonly operated joints, such as hips, ankles, and MTP1. Thus, broadening MSUS protocols to include replaced joints and OJA would increase the sample size in future cohorts. In addition, the operations performed on joint areas without replacement (OJA), were not analyzed based on subtypes due to small sample size. Lastly, our study did not have information on whether the replaced joint or OJA had evidence of prior osteoarthritis that could have acted as a driver of inflammation in replaced joints. Therefore, it is of interest to validate the trends seen in this study with larger cohorts.

## Conclusions

In conclusion, ultrasound is a powerful tool for identifying new or persistent synovitis in joints having underwent surgery, which had yet to be explored systematically in previous studies. Furthermore, the ultrasound-detected response to therapy seen in these joints holds promise for refining therapeutic management of RA patients.

## Data Availability

The datasets used and/or analyzed during the current study are available from the corresponding author on reasonable request.

## Abbreviations

ACR: American College of Rheumatology
CCP: Cyclic citrullinated peptide
CDAI: Clinical disease activity index
DAS: Disease activity score
DMARD: Disease-modifying anti-rheumatic drugs
ESR: Erythrocyte sedimentation rate
FDG-PET: Fluorodeoxyglucose positron emission tomography
GSUS: Grayscale ultrasound
IP: Interphalangeal
IQR: Interquartile range
MCP: Metacarpophalangeal
MSUS: Musculoskeletal ultrasound
MTP: Metatarsophalangeal
OJA: Operations of joint areas
PDUS: Power doppler ultrasound
PIP: Proximal interphalangeal
RA: Rheumatoid arthritis
SD: Standard deviation
US: Ultrasound
